# Characterization of Cortical Glial Scars in the Diisopropylfluorophosphate (DFP) Rat Model of Epilepsy

**DOI:** 10.3389/fcell.2022.867949

**Published:** 2022-03-16

**Authors:** Meghan Gage, Megan Gard, Thimmasettappa Thippeswamy

**Affiliations:** ^1^ Department of Biomedical Sciences, College of Veterinary Medicine, Iowa State University, Ames, IA, United States; ^2^ Neuroscience Interdepartmental Program, Iowa State University, Ames, IA, United States

**Keywords:** astrocytes, microglia, glial scar, epilepsy, chrondrotin sulfate proteoglycans (CS-56), CD68

## Abstract

Glial scars have been observed following stab lesions in the spinal cord and brain but not observed and characterized in chemoconvulsant-induced epilepsy models. Epilepsy is a disorder characterized by spontaneous recurrent seizures and can be modeled in rodents. Diisopropylfluorophosphate (DFP) exposure, like other real-world organophosphate nerve agents (OPNAs) used in chemical warfare scenarios, can lead to the development of *status epilepticus* (SE). We have previously demonstrated that DFP-induced SE promotes epileptogenesis which is characterized by the development of spontaneous recurrent seizures (SRS), gliosis, and neurodegeneration. In this study, we report classical glial scars developed in the piriform cortex, but not in the hippocampus, by 8 days post-exposure. We challenged both male and female rats with 4–5 mg/kg DFP (s.c.) followed immediately by 2 mg/kg atropine sulfate (i.m.) and 25 mg/kg pralidoxime (i.m.) and one hour later by midazolam (i.m). Glial scars were present in the piriform cortex/amygdala region in 73% of the DFP treated animals. No scars were found in controls. Scars were characterized by a massive clustering of reactive microglia surrounded by hypertrophic reactive astrocytes. The core of the scars was filled with a significant increase of IBA1 and CD68 positive cells and a significant reduction in NeuN positive cells compared to the periphery of the scars. There was a significantly higher density of reactive GFAP, complement 3 (C3), and inducible nitric oxide synthase (iNOS) positive cells at the periphery of the scar compared to similar areas in controls. We found a significant increase in chondroitin sulfate proteoglycans (CS-56) in the periphery of the scars compared to a similar region in control brains. However, there was no change in TGF-β1 or TGF-β2 positive cells in or around the scars in DFP-exposed animals compared to controls. In contrast to stab-induced scars, we did not find fibroblasts (Thy1.1) in the scar core or periphery. There were sex differences with respect to the density of iNOS, CD68, NeuN, GFAP, C3 and CS-56 positive cells. This is the first report of cortical glial scars in rodents with systemic chemoconvulsant-induced SE. Further investigation could help to elucidate the mechanisms of scar development and mitigation strategies.

## Introduction

Glial scars in the spinal cord, following traumatic injury, have been described for decades (DB., 1930; Wanner et al., 2013). Progress in research techniques has allowed for the characterization of these scars. Typically, the spinal cord scars consist of two major parts: the scar core, which contains macrophages and fibroblasts; and the scar periphery, which consists of surrounding reactive astrocytes (Silver and Miller, 2004; Wang et al., 2018). These scars have been primarily studied with respect to their inhibitory effect on axon regeneration at the site of injury (Sugar and Gerard, 1940; Fawcett and Asher, 1999). For many years, these scars were thought to be barriers to recovery after spinal cord injury, but some recent studies suggest that they may be protective (Anderson et al., 2016; Yang et al., 2020). Importantly, the most persuasive argument is that the scar periphery prevents the spread of neuroinflammation, thereby protecting the undamaged tissue (Voskuhl et al., 2009; Sofroniew, 2015). Although most glial scar studies focus on spinal cord injury, there has been some description of scar pathology in the brain following traumatic brain injury as well as stroke (Yutsudo and Kitagawa, 2015; Frik et al., 2018; Schiweck et al., 2021). Importantly, some studies do describe “glial scarring”, usually referring to a massive infiltration and activation of both microglia and astrocytes, but such scarring pathology does not provide evidence to fit the classical definition of “glial scar”, i.e., scar core and scar periphery.

The molecular markers found in glial scars are also known to play a role in epilepsy. For example, increased proliferation and activation of glial cells (astrocytes and microglia) and loss of neurons has been shown in both human and animal models of epilepsy (Farrell et al., 2017; Rana and Musto, 2018). Similarly, glial scars usually have an upregulated expression of transforming growth factor beta (TGFβ) and proteoglycans (Lagord et al., 2002; Hussein et al., 2020) which are also implicated in epileptogenesis (Bar-Klein et al., 2014; Yutsudo and Kitagawa, 2015). TGFβs are pleiotropic cytokines and some studies have shown that they may be responsible for the initiation of glial scar formation (Buss et al., 2007). Proteoglycans are thought to inhibit neurite growth post spinal cord injury by altering the extracellular matrix (McKeon et al., 1999). These commonalities imply that glial scars may be prevalent in epilepsy, though the classical glial scars as presented in this study have not yet been reported in most models.

In this study, we are the first to describe a characteristic glial scar observed 8 days after the induction of *status epilepticus* (SE) in a rat model of systemic chemoconvulsant-induced epilepsy. Epilepsy is the fourth most prevalent neurological disease and is characterized by spontaneous recurrent seizures, gliosis, and neurodegeneration (Fisher et al., 2005; Hirtz et al., 2007; Todorovic et al., 2012; Beghi and Hesdorffer, 2014; Pitkänen and Engel, 2014; Stafstrom and Carmant, 2015; Seinfeld et al., 2016). Despite many available therapeutic agents, approximately 30% of people with epilepsy develop pharmacoresistance; this underscores the importance of studying epilepsy and the discovery of new therapeutic drugs (French, 2007; Engel, 2014). This study aimed to characterize SE-induced glial scars using several molecular markers previously known to play a role in glial scarring after spinal cord injury (glial and neuronal markers, TGFβs, proteoglycans, fibroblasts). We also characterized astrocytic expression of complement 3 (C3), which has previously been used as a marker for astrocytic activation and inducible nitric oxide synthase (iNOS), which is indicative of oxidative stress and neurotoxicity (Garry et al., 2015; Liddelow et al., 2017). Importantly, we previously showed that pharmacological inhibition of iNOS by 1400 W led to reduced gliosis, neurodegeneration, and seizure frequency in a rodent model of diisopropylfluorophosphate (DFP)-induced epilepsy (Putra et al., 2020a).

In this study, we utilized the rat DFP model of epilepsy. We have previously demonstrated that DFP-induced SE, depending on the initial severity, promotes the development epilepsy (Gage et al., 2021a; Putra et al., 2020b). The features of DFP-induced epilepsy are similar to other chemoconvulsant models of epilepsy, such as kainate/pilocarpine model, with respect to the onset of neuroinflammation, neurodegeneration, and spontaneous recurrent seizures (SRS) (Curia et al., 2008; Putra et al., 2020a; Sharma et al., 2021). DFP is an irreversible inhibitor of acetylcholinesterase, leading to accumulation of acetylcholine and overstimulation of cholinergic receptors (McDonough and Shih, 1997; Millard et al., 1999; Todorovic et al., 2012; Puttachary et al., 2016; Gage et al., 2020). DFP is used to model the effects of other organophosphate nerve agents (OPNAs) such as Soman and Sarin, which have been previously used as chemical weapons (Morita et al., 1995; Suzuki et al., 1995; Okumura et al., 1996; Miyaki et al., 2005; Yanagisawa et al., 2006). OPNAs or DFP exposure leads to the development of cholinergic crisis, which includes symptoms such as bronchoconstriction, salivation, lacrimation, gastrointestinal distress, bradycardia, and convulsions or SE (McDonough and Shih, 1997; Jett, 2012). In this study, we challenged animals with DFP and observed glial scars in the amygdala/piriform cortex, which look like those observed post-stab injury to the brain or spinal cord. This study aimed to determine the prevalence of these scars as well as to characterize the cell types located at both the scar core and scar periphery.

## Methods

### Animals, Care and Ethics

25 male and female Sprague Dawley rats were purchased from Charles River (Wilmington MA, United States). Fifteen (7 male, 8 female) animals were challenged with DFP, and 10 animals (5 male, 5 female) were left untreated as controls. All animals were sacrificed with 100 mg/kg pentobarbital sodium (i.p.) purchased from the Lloyd Veterinary Medical Center Hospital Pharmacy, Ames, Iowa. The procedures were approved by the Institutional Care and Use Committee (IACUC-21-109) at Iowa State University. Animals were single housed with 12-h light and dark cycles and given *ab libitum* access to food and water.

### Chemicals

Diisopropylfluorophosphate (Sigma-Aldrich, purity >97%) was prepared fresh in cold phosphate-buffered saline (PBS) prior to administration. Atropine sulfate (ATS, Thermo-Fisher Scientific) and 2-pralidoxime (2-PAM) were prepared fresh in saline. Midazolam (MDZ) was purchased from the Lloyd Veterinary Medical Center Hospital Pharmacy. Gelatin for tissue embedding consisted of 15% type A porcine gelatin, 7.5% sucrose, and 0.1% sodium azide. Citric acid buffer contained 10 mM citric acid and 0.05% tween-20 at a pH of 6. Blocking buffer consisted of 10% donkey serum and 0.05% tritonX-100 in PBS. Antibodies used for immunohistochemistry (IHC) are summarized in Supplementary Table S1. All antibodies were tested with a negative control simultaneously, and the optimal concentration was determined by serial dilution. Antibodies were diluted in PBS containing 2.5% donkey serum, 0.1% tritonX-100 and 0.25% sodium azide. Streptavidin conjugated antibodies were diluted in PBS.

### Seizure Induction and Seizure Scoring

**FIGURE 1:**
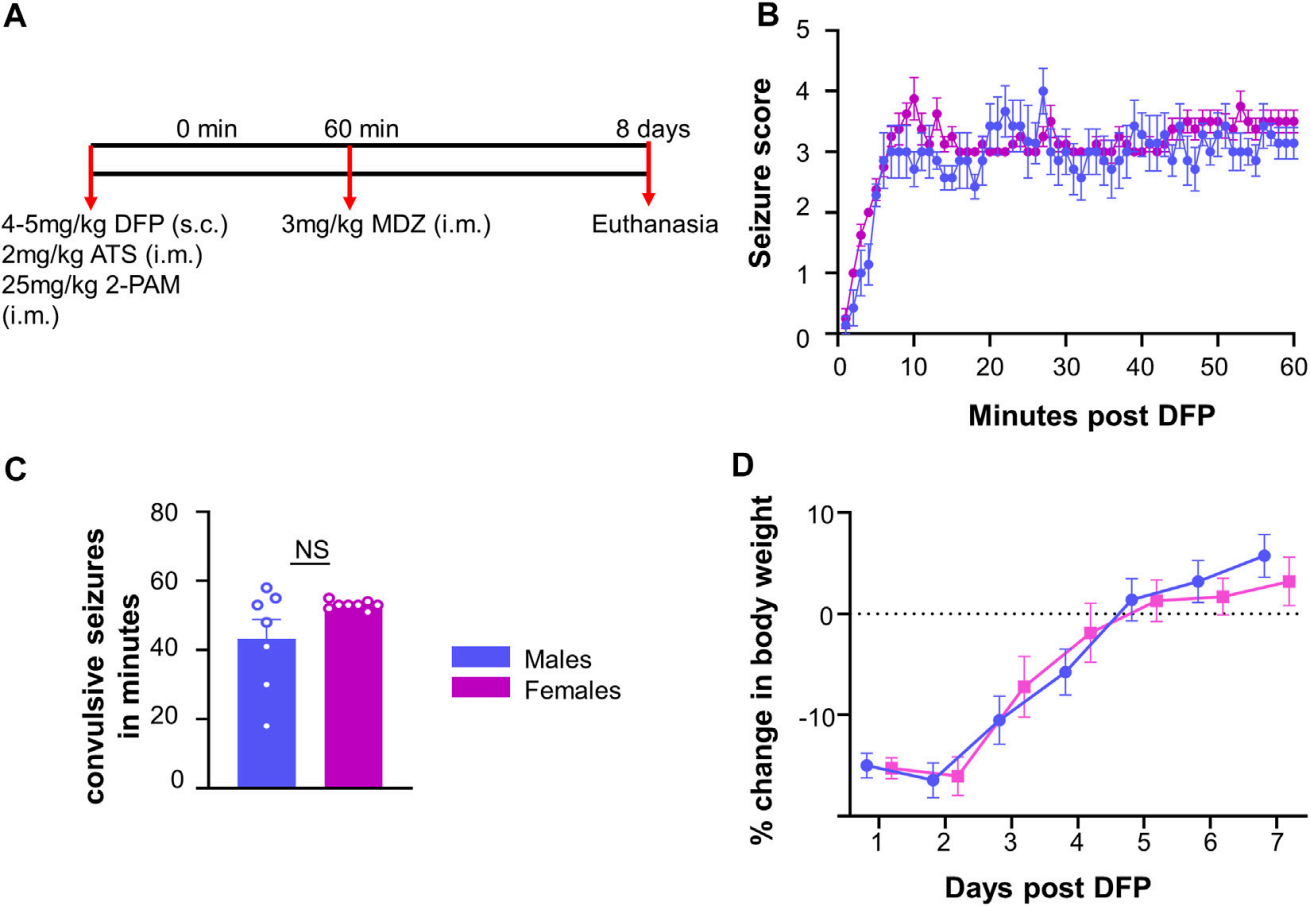
Experimental outline for challenge with diisopropylfluorophosphate (DFP). **(A)** Male and female rats were injected with 4–5 mg/kg DFP (s.c.) followed immediately by 2 mg/kg atropine sulfate (ATS, i.m.) and 25 mg/kg 2-PAM (i.m.). One hour later, behavioral seizures were terminated with 3 mg/kg midazolam (MDZ, i.m.). **(B)** Average seizure stage each minute following DFP (mixed measures ANOVA, *n* = 7–8). **(C)** Number of minutes in a CS prior to MDZ administration; **(D)** Average change in body weight after DFP intoxication. *t*-test or mixed measures ANOVA (*n* = 7–8).

The experimental design is illustrated in Figure 1A. Fifteen rats (7 males and 8 females) were challenged with 4 mg/kg (males) or 5 mg/kg (females) DFP (s.c.) followed by 2 mg/kg ATS (i.m.) and 25 mg/kg 2-PAM (i.m.) to reduce mortality. In our previous studies, female rats required a higher dose of DFP than males when they were challenged independently (Gage et al., 2020). Animals developed seizures within 5–10 min after DFP, and one hour later, MDZ (3 mg/kg, i.m.) was administered.

Prior to MDZ, animals were scored on each minute for seizure stage based on a modified Racine scale (Racine, 1972) as described in our previous publications (Putra et al., 2020b; Gage et al., 2021b; Sharma et al., 2021). Stage one was characterized by salivation, lacrimation, urination, defecation, and mastication, while stage two was characterized by head nodding and tremors. Stage three was characterized by rearing, Straub tail, and forelimb extension. Stage four presented with the loss of the righting reflex and forelimb clonus, while stage five included repeated rearing, falling, and circling. To measure seizure duration and severity, we calculated the number of minutes in which each animal was in a convulsive seizure (CS) (stage 3–5); stages 1 and 2 were considered nonconvulsive seizures (NCS).

### Immunohistochemistry

Animals were sacrificed 8 days after DFP exposure. Transcardiac perfusion with PBS followed by 4% paraformaldehyde was performed to preserve the morphology. Brains were dissected and incubated for 24 h in 4% paraformaldehyde followed by 25% sucrose (in PBS) for 72 h in a refrigerator. Brains were embedded in gelatin, rapidly frozen in liquid nitrogen cooled isopentane, and stored at −80°C. Coronal brains were then sectioned serially using a cryostat (ThermoFisher) into 16 μm sections beginning at the most rostral portion of the hippocampus. Each slide contained sections approximately 480 μm apart to cover regions of the brain from rostral to caudal. Slides were stored at −20°C and once stained, stored at 4°C.

Slides were subjected to antigen retrieval in citric acid buffer at 95°C for 23 min. After transferring to Shandon racks, slides were washed with PBS for one hour and then incubated in blocking buffer for one hour. Slides were incubated with the desired primary antibodies overnight at 4°C. The next day, slides were again washed with PBS for one hour and incubated with the appropriate biotinylated, alexaflour, or FITC-conjugated secondary antibodies. Slides were again washed with PBS for one hour prior to incubation with streptavidin-conjugated antibodies. After another hour wash in PBS, slides were mounted with a medium containing DAPI (Vectashield).

### Cell Quantification

**FIGURE 2:**
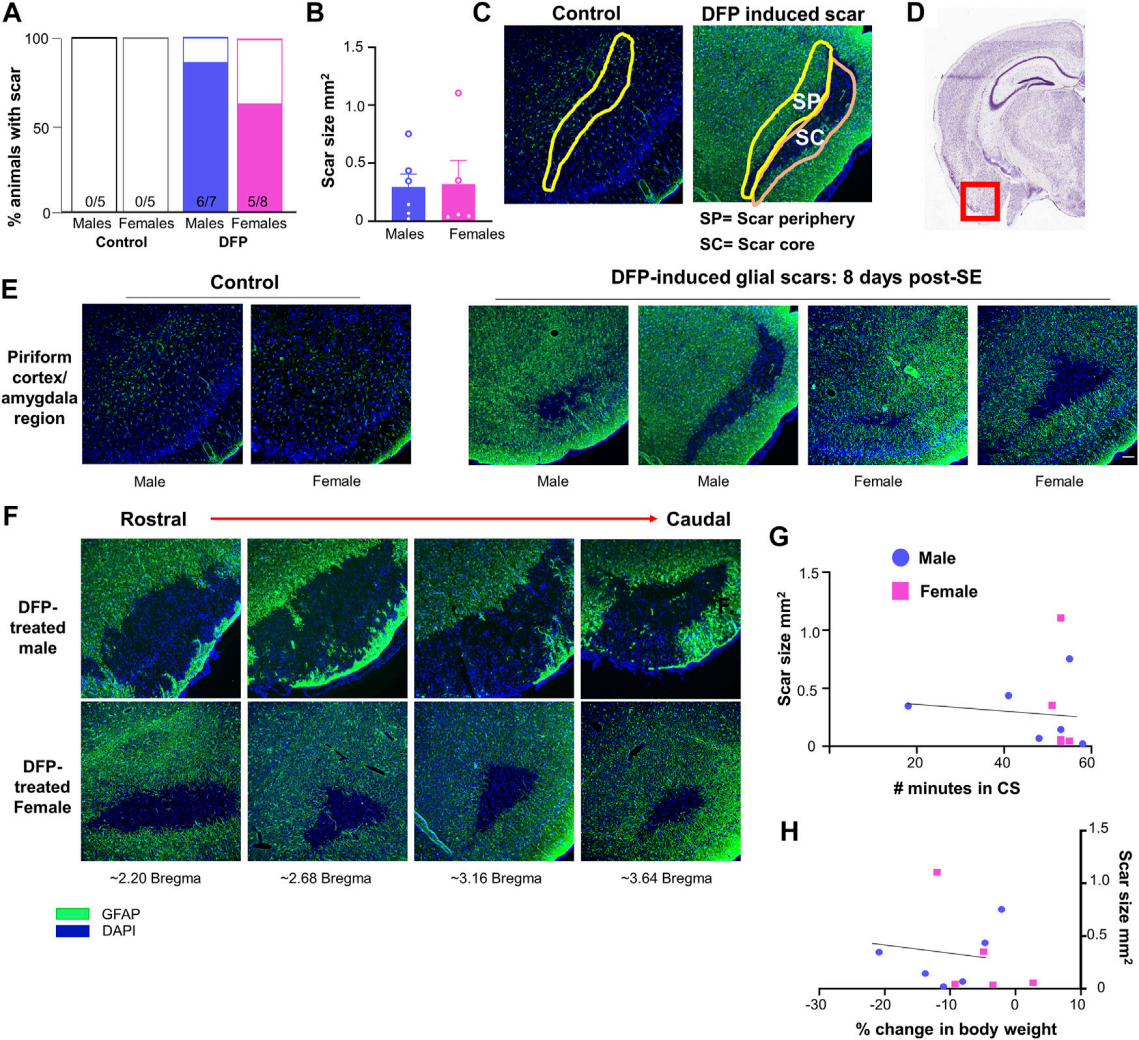
Glial scar prevalence, shape, and size. **(A)** Percent of animals displaying a glial scar in male and female rats. **(B)** Average scar area comparison between male and females across sections. **(C)** Example of areas quantified in the scar core (SC) and scar periphery (SP). A similar region was analyzed in control animals. **(D)** Region imaged relative to the rest of brain (Image courtesy: Allen brain atlas, United States) **(E)** Representations of the variability in scar shape and size in the piriform cortex/amygdala region. **(F)** Scars often extended over multiple sections; images are from the same animal in the amygdala/piriform cortex region spreading from rostral to caudal. **(G, H)** Relationship between scar size and the number of minutes in a convulsive seizure (CS) during SE or the loss in body weight over the first three days post-DFP.

The Lecia DMi8 inverted fluorescence microscope and Leica K5 passive cooled sCMOS camera were used to image the slides. Images were taken from at least four sections per animal for each marker. For animals with glial scars, images were taken only from sections containing the scar. For controls without glial scars, images were taken from a similar region to the scar location (amygdala/piriform cortex region). Values were averaged across the sections. ImageJ was used to isolate the desired regions of the brain (scar core and scar periphery) based upon the localization of GFAP staining. The scar core was defined as the GFAP negative region amidst intensely stained GFAP cells in the periphery. The scar periphery (200–350 μm) was defined as the inner region adjacent to the scar core (represented in Figure 2C) since the outer region of the scar was close to the surface of the brain/the pyramidal cell layer. We chose this layer rather than the outer layer as some of the scars did not have an intact outer layer of GFAP positive cells (for example, Figure 2E). A similar area was isolated in controls without scars. ImageJ was also used to measure the area of the scars; area was averaged between sections. ImageJ and cell profiler software were used to quantify various molecular markers in the brain. The general parameters used for each marker are described in Supplementary Table S2.

### Statistics and Rigor

GraphPad Prism (version 9.3.0) was used to graph the data and perform various statistical analyses. Where appropriate, experimenters were blind to treatment groups. Normality was assessed *via* the Shapiro-Wilk test, and Grubb’s test was used to eliminate outliers. Linear models and regression analyses were used to analyze the data and included both treatment and sex as a variable. Sex was analyzed within treatment groups and treatment was analyzed within region. Specific statistical tests can be found in the corresponding figure legends.

## Results

### Initial Seizure Response to DFP and Glial Scar Prevalence

Following DFP intoxication, both male and female animals developed CS in 5–10 min. The average seizure stage at each minute is represented in Figure 1B. There was no significant difference in the amount of time male and female animals spent in CS during SE although females received 1 mg/kg more DFP than males (Figure 1C). However, the females had a less variable response than males which could be due to the higher dose of DFP. We considered SE severity as a criterion, irrespective of the dose of DFP, to compare the prevalence of the glial scars between sexes (Figure 1C).

Glial scars were recognized by intense GFAP positive cells (astrocytes) concentrated around a GFAP negative brain region in the piriform cortex and amygdala regions in both males and females (Figures 2D,E). Located just deep to the pyramidal cell layer of the piriform cortex, astrocytes formed the borders around a “hole-like” structure, the periphery of glial scar as described for the spinal cord injury models. We did not observe the glial scars in other regions of the brain. No control animals (not treated with DFP) had any glial scars while 6/7 DFP treated males and 5/8 DFP treated females had a glial scar in at least one section 8 days after exposure to DFP (Figure 2A). There was variability in the size of the scar though they were typically “bean shaped” as shown in Figure 2E. There was no difference in the size of the scar between sexes (Figure 2B). Scars spanned from the rostral to caudal end of the brain in both sexes (Figure 2F **)**. DFP treated animals, irrespective of sex, lose bodyweight for the first three days (Gage et al., 2021a). In this study too, both sexes lost weight but recovered to their basal levels by day 5 (Figure 1D). There was no significant correlation between scar size and initial SE duration or change in body weight in the first three days (Figures 2G,H).

### Microglia, CD68 Positive Phagocytic Microglia, and Neurons in and Around the Scar

**FIGURE 3:**
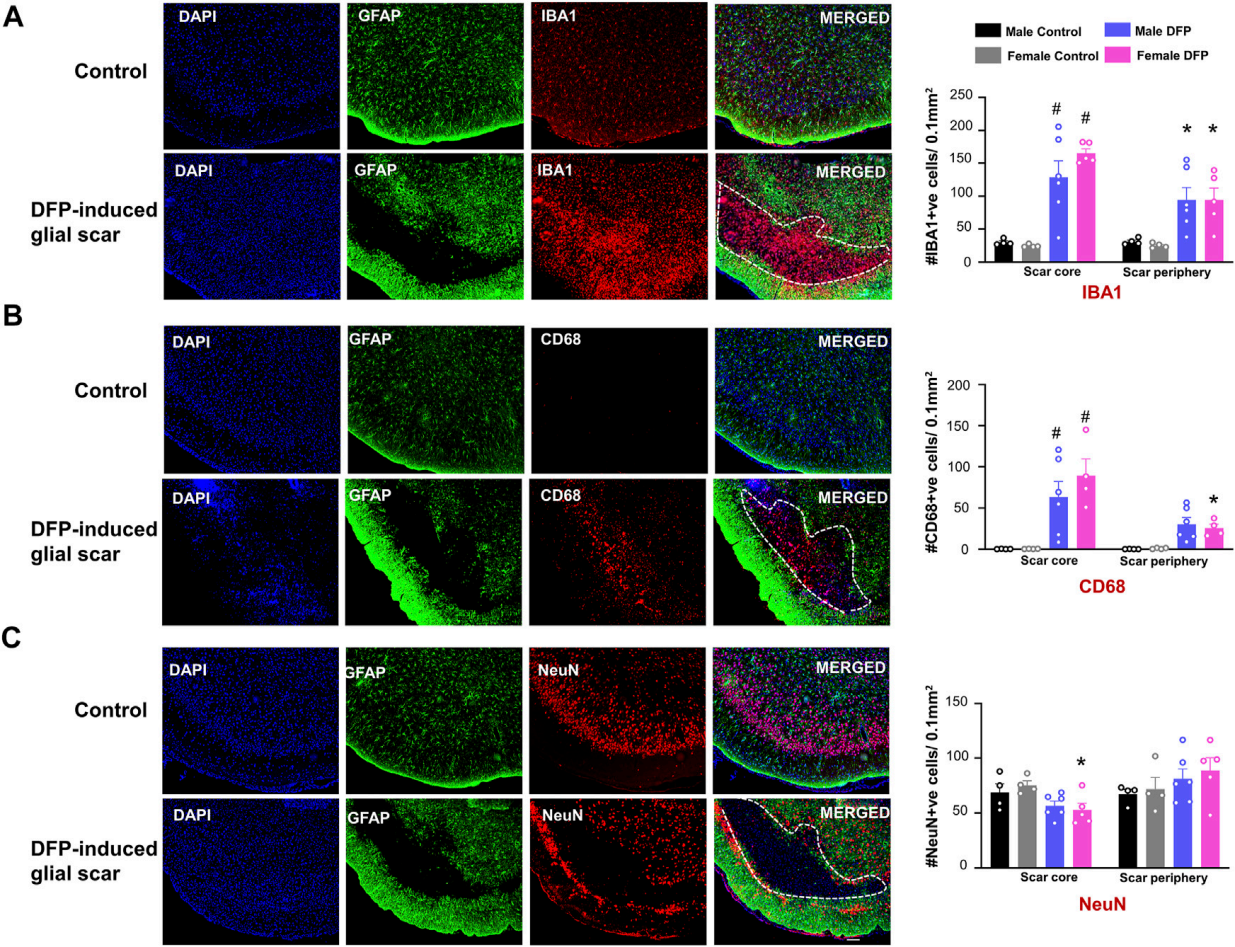
Characterization of the glial scars. We compared the cell markers in the scar core with the scar periphery. **(A)** Number of IBA1 positive microglia and macrophages **(B)** Number of CD68 positive phagocytic cells **(C)** Number of NeuN positive cells. Mixed measures ANOVA with Tukey post-hoc, *n* = 4–6, *p < 0.05 comparison of sex matched “scar core versus scar periphery”, #<*p*< 0.05 compared to sex matched control in the same region. Scale = 100 μm.

For DFP treated animals with scars, we quantified the cell types in the region inside the scar (scar core) and a similar surface area of a region outside the scar (scar periphery), as shown in Figure 2C. Images are taken from the same animal. To verify that the changes in cellular density are not due to differences in cortical regions, we also analyzed two similar regions in control animals. For controls, the “scar core equivalent” was a region just adjacent to the pyramidal cell layer while the “scar periphery equivalent” was a region further deep to the “scar core equivalent”. We used a linear model to compare treatment, location (scar core versus periphery), and sex. In the scar region of DFP-treated males and females, compared to the controls, there were significantly more IBA1-positive and CD68-positive microglia cells (Figures 3A,B). In DFP treated animals, in both sexes, there was a significant upregulation of IBA1 positive cells in the scar core compared to the periphery (Figure 3A). There was also an upregulation of CD68 positive cells in the scar core compared to the periphery in DFP treated animals, but the upregulation was statistically significant in females only (Figure 3B). In DFP treated females, there was also a statistically significant reduction in the number of NeuN positive cells in the scar core compared to the scar periphery, but in males, the differences were not significant (Figure 3C). Interestingly, when DFP-treated animals were compared with their respective sex-matched controls, there were no significant differences in NeuN positive cells in either the scar core or the scar periphery (Figure 3C). In controls, there was no change in either IBA1, CD68, or NeuN positive cells between scar core or peripheral equivalent regions. When sex was directly compared within each region (scar core or scar periphery in DFP treated groups and scar core equivalent or scar periphery equivalent in controls) there were no statistical differences (Figures 3A–C).

### GFAP and C3 Positive Cells

**FIGURE 4:**
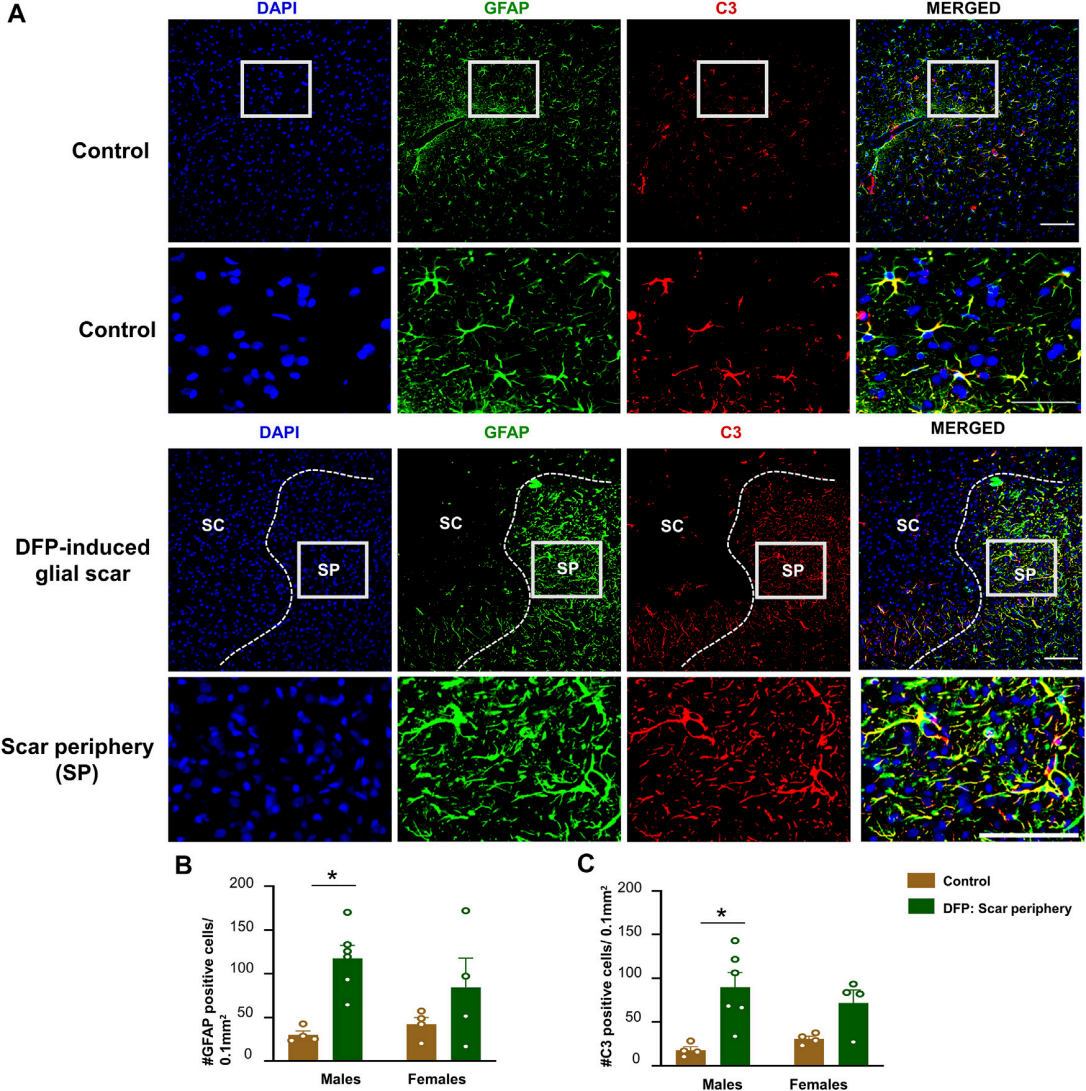
Characterization of the glial scar cells outside the core. **(A)** Representative images for GFAP and C3 staining in control animals and DFP treated animals with glial scars. We compared the cell counts in the scar periphery to a similar region in controls. **(B)** Number of GFAP positive cells. **(C)** Number of C3 positive cells. Mixed measures ANOVA, *n* = 4–6, *p < 0.05. Scale = 50 μm.

The core of the glial scar had no GFAP positive astrocytes. Hypertrophic astrocytes surrounded the core of the glial scar **(** Figure 4A). There was an upregulation of astrocytes (GFAP positive cells) in the scar periphery compared to a similar area in untreated sex-matched control animals (Figure 4B). This was significant in males but only trending in females. We co-stained sections with GFAP and C3, a complement component marker, which is also a marker for astrocytic activation (Liddelow et al., 2017; Putra et al., 2020a). Both control animals and DFP treated animals had astrocytic C3 positive staining, but there was a significant increase in the number of GFAP and C3 positive cells in a male DFP treated animals with scars compared to male control animals (Figures 4B,C). Again, this was only trending in females. There were no statistically significant sex differences in GFAP or C3 positive cells (Figures 4B,C).

### TGF-β Positive Cells

**FIGURE 5:**
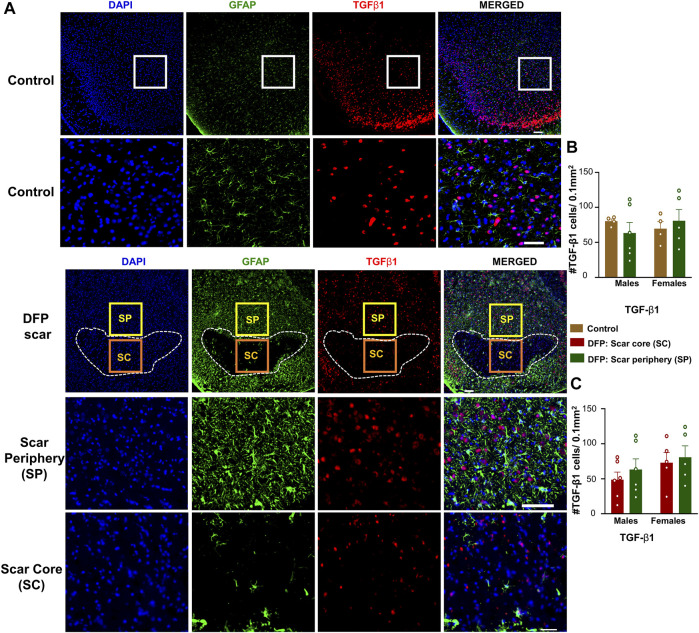
TGF-β1 signaling in control animals and DFP treated animals with glial scarring. **(A)** Representative images for GFAP and TGF-β1. **(B)** The number of TGF-β1 positive cells in the scar periphery (SP) were compared to a similar region in controls. **(C)** Comparison of the number of TGF-β1 positive cells in the scar periphery and the scar core (SC). Mixed measures ANOVA, *n* = 4–6, *p < 0.05. Scale = 50 μm.

**FIGURE 6:**
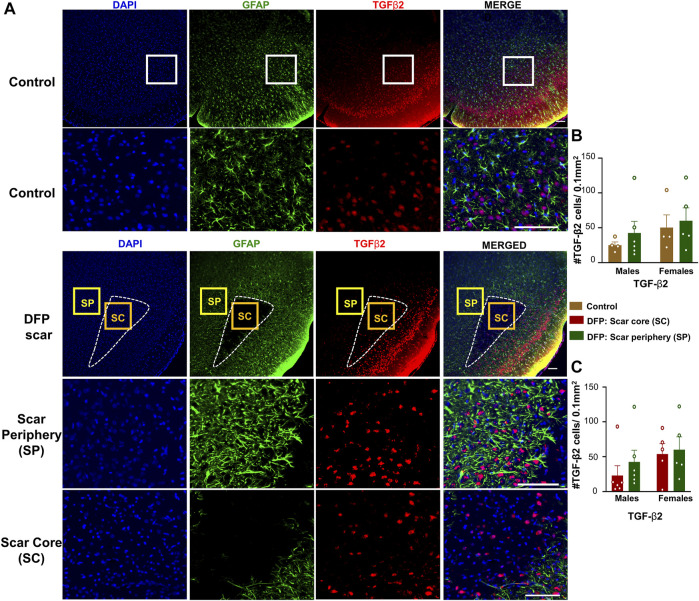
TGF-β2 signaling in control animals and DFP treated animals with glial scarring. **(A)** Representative images for GFAP and TGF-β2. **(B)** The number of TGF-β2 positive cells in the scar periphery (SP) were compared to a similar region in controls. **(C)** Comparison of the number of TGF-β2 positive cells in the scar periphery and the scar core (SC). Mixed measures ANOVA, *n* = 4–6, *p < 0.05. Scale = 50 μm.

TGF-β1 and TGF-β2 regulate both inflammation as well as the extension of neurites at the vicinity of glial scars (Shiying et al., 2017). We hypothesized that the density of TGF-β positive cells in the scar periphery might be different from the scar core or from control animals (Figure 2C). TGF-β1and TGF-β2 immunopositive cells did not colocalize with GFAP and were present in both controls, and DFP treated animals (Figures 5A, 6A). There was no significant difference between the DFP treated animals (scar periphery) and control animals in the number of TGF-β1 or TGF-β2 positive cells (Figure 5B, Figure 6B **)**. There was also no difference in the of TGF-β1 or TGF-β2 positive cells in the scar periphery compared to the scar core in DFP treated animals (Figure 5C, Figure 6C). There were no significant sex differences in the number of TGF-β1 or TGFβ-2 positive cells either (Figure 5B,C, Figure 6B,C).

### iNOS Positive Cells

**FIGURE 7:**
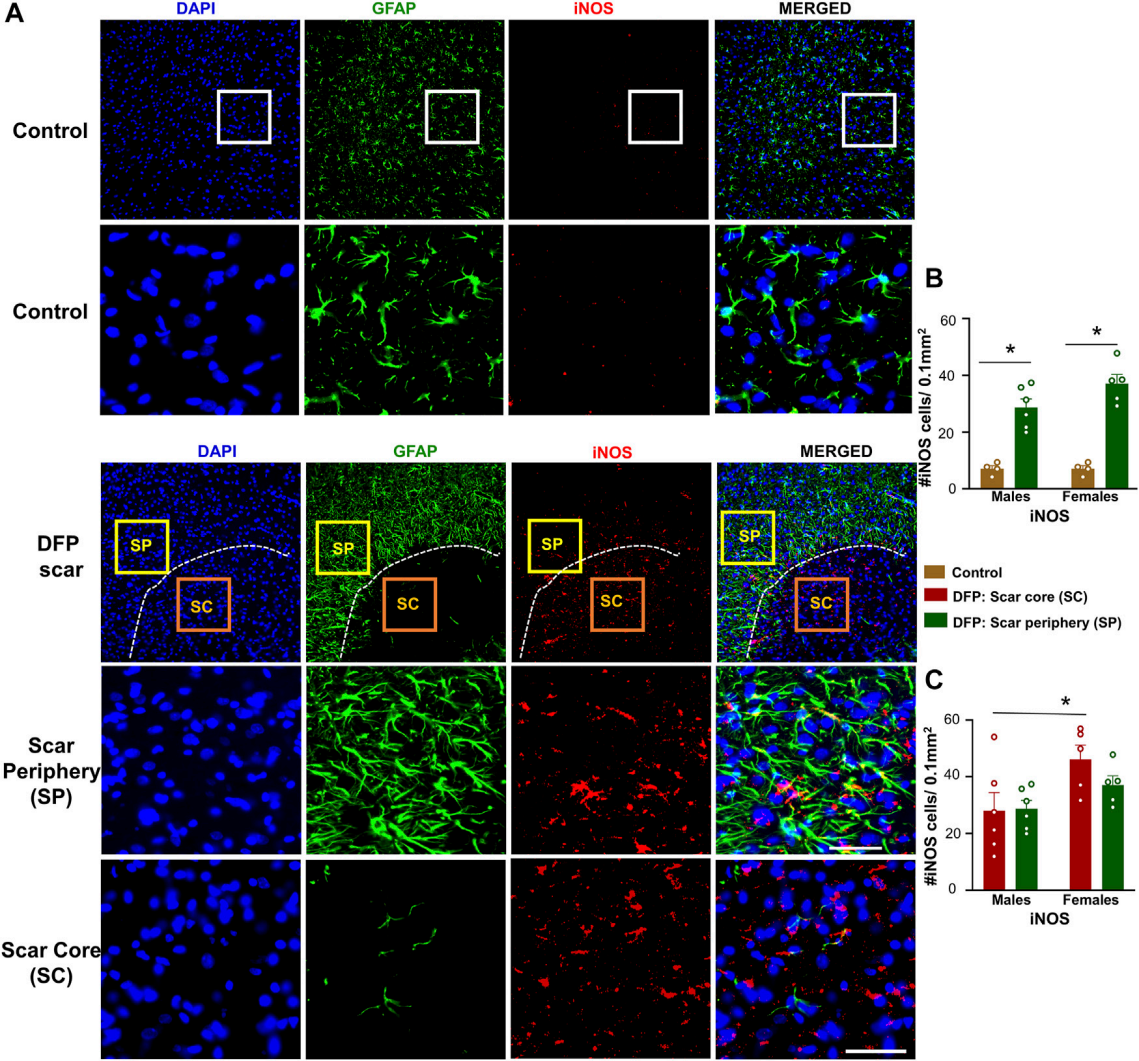
Inducible nitric oxide synthase (iNOS) signaling in control animals and DFP treated animals with glial scarring. **(A)** Representative images for GFAP and iNOS. **(B)** The number of iNOS positive cells in the scar periphery (SP) were compared to a similar region in controls. **(C)** Comparison of the number of iNOS positive cells in the scar periphery and the scar core (SC). Mixed measures ANOVA, *n* = 4–6, *p < 0.05. Scale = 50 μm.

Since we found a significant neuronal loss in glial scar, we hypothesized that iNOS may be relevant to glial scars. Representative images of GFAP (to visualize the scars) and iNOS positive staining are shown in Figure 7A. There was a significant upregulation of iNOS positive cells in the scar periphery compared to a similar region in controls (Figure 7B). There were no significant differences between the density of iNOS positive cells in the scar periphery versus the scar core (Figure 7C). Within the scar core, females had significantly more iNOS positive cells compared to males (Figure 7C), which corroborates with a significant reduction in NeuN positive cells (Figure 3C).

### Characteristic Glial Scar Markers: Chromatin Sulfate Proteoglycans (CS-56) and Fibroblasts (Thy 1.1)

**FIGURE 8:**
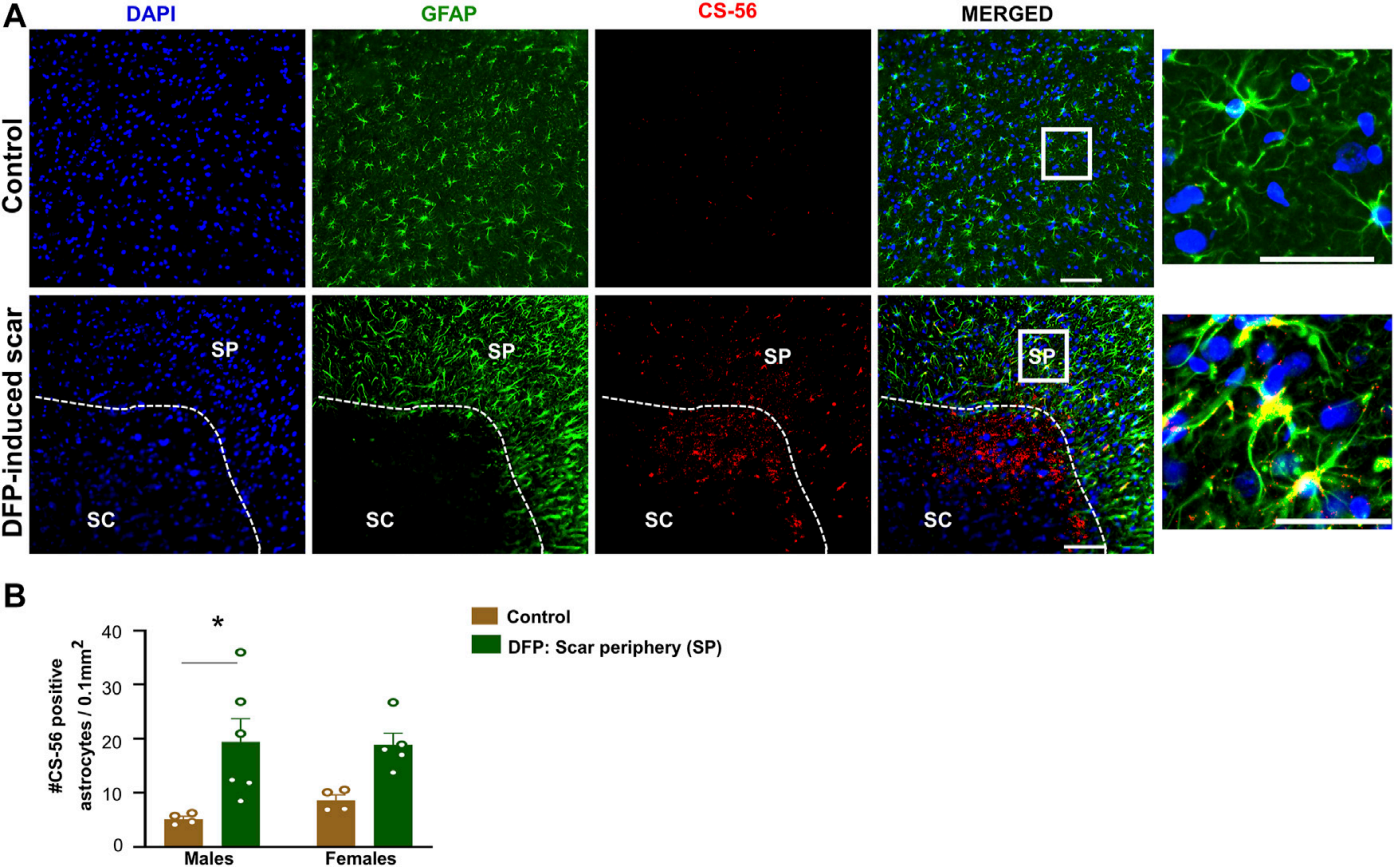
Chromatin sulfate proteoglycan (CS-56) and GFAP staining. **(A)** Representative images for GFAP and CS-56. **(B)** CS-56 positive astrocytes in the scar periphery (SP) were compared to a similar region in controls. Mixed measures ANOVA, *n* = 4–6, *p < 0.05. Scale = 50 μm.

**FIGURE 9:**
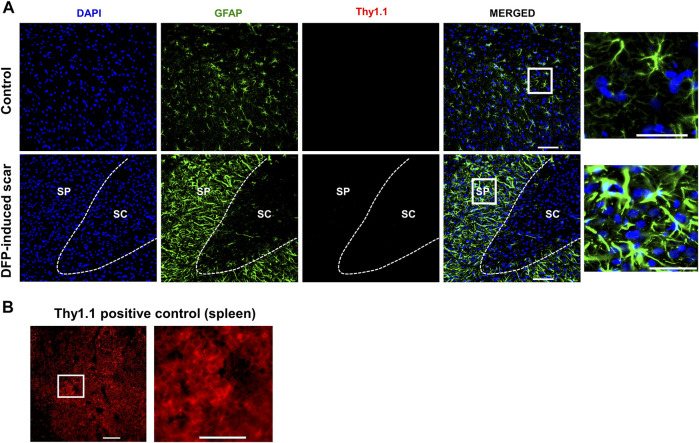
No fibroblasts (Thy1.1) in the scar region. **(A)** There was no Thy 1.1 positive staining in the scar periphery (SP) or scar core (SC) DFP-induced glial scars or in controls. **(B)** Thy1.1 positive staining in the spleen. Scale = 50 μm.

Proteoglycans and fibroblasts are highly associated with glial scars in spinal cord injury (Silver and Miller, 2004; Properzi et al., 2005). In DFP treated males and females with glial scars, CS-56 was observed in both core and the periphery. In DFP treated males, compared to sex matched controls, there was a significant increase in positive staining for CS-56, which indicates the presence of chondroitin sulfate proteoglycans (CSPG) (Figures 8A,B). The same trend was observed in females, but it was not significant. CS-56 cells were primarily co-localized with astrocytes (GFAP positive cells) at the scar periphery**.** We did not detect Thy 1.1 fibroblasts either in the DFP treated animals or controls in the specified areas **(** Figure 9A). We used sections of spleen tissue (untreated rat) as a positive control for Thy1.1 staining (Figure 9B).

## Discussion

This is the first study to report the presence of glial scars in the brain following DFP-induced epilepsy. Glial scars have long since been reported in the spinal cord following traumatic injury (Silver and Miller, 2004; Leal-Filho, 2011; Yuan and He, 2013). For many years, these scars were thought to be barriers to axonal regeneration. However, current evidence suggests that glial scars can be protective by limiting the spread of injury (Rolls et al., 2009; Cregg et al., 2014; Yang et al., 2020)**.** Though far more studied in spinal cord injury, these types of glial scars have also been described in traumatic brain injury, ischemia, and multiple sclerosis (Robel et al., 2015; Frik et al., 2018; Luo et al., 2018; Wang et al., 2018). Some studies have associated the glial scars with post-traumatic epilepsy (Robel, 2017; Xu et al., 2019). Another study reported a similar glial scar 15 days post kainate (KA) injection directly into the rat thalamus (Dusart et al., 1991). Although the latter KA model is an acquired epilepsy, our study is different in that the epileptic insult arose from a systemic chemical insult (DFP). In this study, we found a high prevalence of scars (73%) following DFP-induced SE. Also interesting, the glial scars were localized to the piriform cortex/amygdala region. Neuropathological changes in these regions, such as neurodegeneration and gliosis, have been reported following OP exposure, but glial scars are not reported (Aroniadou-Anderjaska et al., 2009, 2016).

There was a significant increase in iNOS positive cells in the female glial scar cores compared to male glial scar cores. In DFP treated females, but not males, there was a significant increase in CD68 positive cells and significant reduction in NeuN positive cells in the scar core compared to the periphery. Compared to sex-matched controls, there was also a DFP-induced increase in GFAP, C3 and CS-56 positive cells that was only significant in males. Also of note, this study relies on behavioral evaluation of seizures rather than a more robust method such as electroencephalography (EEG). Electrode implantation could lead to changes in neuroinflammation and possibly influence the glial scarring, which can confound the real effects of systemic chemoconvulsant induced glial scars (Tse et al., 2021). We have previously demonstrated that these behaviors are strongly associated with EEG changes and thus, visual behavioral seizure scoring by an experienced experimenter is a reliable measure of initial SE severity (Putra et al., 2020a; Putra et al., 2020b).

It is well established that epilepsy is associated with abnormal regulation and excessive proliferation of glial cells (Vezzani et al., 2015). Our previous studies and others’ studies have demonstrated that DFP-induced epilepsy is also characterized by neuroinflammation and neurodegeneration (Gage et al., 2021a; Gage et al., 2021b; Guignet et al., 2019; Putra et al., 2020a). Reactive astrogliosis exists on a spectrum ranging from hypertrophy of astrocytes and progressing into the formation of a glial scar (Sofroniew, 2005; Pekny and Pekna, 2016). The roles of astrocytes in both the normal and epileptic brains are becoming more diverse. Historically, astrocytes are associated with the formation of the blood-brain barrier, metabolic processes, neurodevelopment, and maintenance of cellular homeostasis (Abbott et al., 2006; Clarke and Barres, 2013; Benarroch, 2016). Reactive astrogliosis has become a hallmark of epileptogenesis, though to our knowledge, no study has described reactive astrogliosis in which these cells form the periphery of a scar following systemic chemical epileptic insult (de Lanerolle et al., 2010; Robel et al., 2015; Verhoog et al., 2020).

Astrocytes surrounded the core of the scar. Compared to controls, these cells were more in number, hypertrophic, and expressed C3, which is considered a marker for reactive astrocytes (Liddelow et al., 2017; Hartmann et al., 2019; Putra et al., 2020b). C3 is part of the complement system, which is an important regulator of chemotaxis, phagocytosis, and cell adhesion (Frank and Fries, 1991). Though it was upregulated in DFP treated animals, there was still C3 positive staining in the control animals, which may suggest that basal levels play a role in normal brain function. One study showed that C3 knockout mice had increased axon regeneration following spinal cord injury implying its inhibitory role in neurite outgrowth (Peterson et al., 2017). In addition, C3 and other complement proteins are also known to play a role in astrocytes and microglia interaction. Following injury, C1q binds to the cell surface, leading to the cleavage of C3; the proteolytic products then bind to various receptors to initiate the activation of phagocytes (Van Beek et al., 2003; Wagner and Frank, 2009). However, it is unknown whether the C3 proteolytic products of the astrocytes surrounding the scar would influence the microglial function in the scar core.

Like the scars in the spinal cord injury, reactive astrocytes in glial scars of the piriform cortex surrounded a region containing large clusters of microglia. These microglia were reactive in their morphology and also expressed CD68, a marker for microglial activation (Di Gregorio et al., 2005). Notably, both microglia and macrophages express both IBA1 and CD68, so it is possible that macrophages were also present due to the disruption of the blood-brain-barrier post-SE (da Fonseca et al., 2014; Ramprasad et al., 1996). In this study, we found a reduction in neurons (NeuN positive cells) in the scar core (compared to the scar periphery in females), indicating neuronal death and clearance by microglia and macrophages (CD68 positive cells). Similarly, there was an increase in iNOS positive cells in the glial scar region implying increased release of NO that causes neurodegeneration (Thippeswamy et al., 2006; Doherty, 2011). It is important to note that there was no significant reduction in neurons in the DFP-treated groups compared to their respective controls (though there was a trend). This requires further investigation, perhaps with a large sample size and SE severity-matched DFP-treated animals. Likely, the astrocytes respond to microglia-induced neuronal injury to form the scar (Van Beek et al., 2003; Wagner and Frank, 2009). Further investigation will reveal the time-dependent progression of glial scar formation development.

TGF-βs are a pleiotropic cytokines that are involved in regulating the growth and differentiation of various cells, immune regulation, and extracellular matrix metabolism (Vivien and Ali, 2006; Dobolyi et al., 2012). TGF-βs elicit responses *via* interaction with TGF-β receptors and subsequent activation of Smad transcription factors (Hiew et al., 2021). In the context of neurological disease, TGF-βs are sometimes shown to be neuroprotective and are downregulated in many models of neurological disease (Tesseur et al., 2006; Vivien and Ali, 2006; Kashima and Hata, 2018). In contrast, some studies show that TGF-βs could be responsible for the initiation of the glial scar post spinal cord injury (Buss et al., 2007; Shiying et al., 2017; Song et al., 2019). TGF-β1 has been reported to be upregulated early in scar formation while TGF-β2 is upregulated at a later stage (Lagord et al., 2002; Silver and Miller, 2004). Interestingly, we did not find a significant upregulation of TGF-β1 or TGF-β2 in the scars of the DFP treated animals compared to controls which might suggest that these scars respond to injury independent of TGF-β signaling or that the signaling plays a role earlier or later into scar development as this study was limited to 8 days post-DFP intoxication.

Glial scars elsewhere are also associated with the upregulation of chondroitin sulfate proteoglycans (CPSGs). CSPGs are one of several proteoglycan families, which perform a wide variety of functions throughout the body related to development, tissue repair, and organization of cellular structure (Couchman and Pataki, 2012; Iozzo and Schaefer, 2015). In the brain, CSPGs are known to be produced by neurons and astrocytes and are upregulated in a variety of neurological disorders, including spinal cord injury, multiple sclerosis, ischemia (Davies et al., 1999; Carmichael et al., 2005; Deguchi et al., 2005; Siebert et al., 2014). One study in an epilepsy model found increased CPSGs in the mouse cortex following kainate administration (i.p); they also showed increased seizure susceptibility in mice overexpressing chondroitin 6-sulfated chains (C6ST-1) (Yutsudo and Kitagawa, 2015). However, the mouse study did not report the occurrence of glial scars in the hippocampus or in the cortex in either C6ST-1 transgenic mice or wild-type C57BL/6 mice. In the rat DFP model, we found an increase of CS-56, a marker of CPSGs, in the astrocytes surrounding the scars. In the context of glial scars, proteoglycans are thought to be inhibitory for neuronal growth, but their function is unclear in DFP-induced neurotoxicity (McKeon et al., 1999). Importantly, the CSPGs consist of a wide family of molecules including, but not limited to, versican, neurocan, aggrecan and brevican, each of which may play different roles during scar development (Avram et al., 2014). Future work could determine the roles of each specific CSPGs as well as other families of proteoglycans.

Classical spinal cord glial scars also contain fibroblasts that originate from meninges, choroid plexus, and perivascular spaces (Dorrier et al., 2021; Silver and Miller, 2004; Xu and Yao, 2021). In the rat DFP study, no trauma was involved. However, we tested for fibroblasts marker, Thy 1.1, since the scar was close to the external surface of the cortex (Phipps et al., 1989). We did not detect Thy1.1 positive cells in the glial scars, likely due to the nature of injury caused by DFP. Traumatic brain injury compromises the blood-brain-barrier integrity; thus, reports suggest fibroblast infiltration into the injured site from the perivascular spaces (Kawano et al., 2012).

The cellular mechanisms of glial scars following spinal cord injury are temporal in nature (Silver and Miller, 2004; Duan et al., 2015). In our 8-days timepoint study, we characterized the cell types of the glial scar. However, it would be interesting to look at a longer timepoint, to see how long these scars persist and whether the molecular markers and cell-cell interactions change over time. Future work could use more advanced imaging techniques to track the presence and size of the scar over time and the impact of interventional drugs. It would also be interesting to characterize these scars in other models of chemoconvulsant-induced epilepsy such as the kainate, pilocarpine, or more potent OPNA models. This might reveal some interesting model differences in glial scar formation.

## Conclusion

This is the first report of a cortical glial scar following systemic chemoconvulsant-induced SE, like those seen after mechanical injury to the brain or spinal cord (Silver and Miller, 2004; Wang et al., 2018). Scars were characterized by a core consisting of large clusters of phagocytic microglia and macrophages, characterized by a significant increase in iNOS and CD68. These phagocytic microglia and iNOS likely led to neurodegeneration which was evidenced by the reduction of NeuN in the scar core, though not statistically significant. The periphery of the scar consisted of hypertrophic, C3, and CSPG expressing astrocytes though there was no change in TGF-β1 or TGF-β2 expression. Future studies could use other glial and neuronal-specific signaling pathway markers to better understand the molecular mechanisms involved in scar formation. Importantly, it is unknown whether these scars are protective or harmful in the context of DFP-induced epilepsy. Future studies could correlate the prevalence or size of these scars with other epileptogenic parameters such as spontaneous seizures, electrographic spikes, or other changes in neurobehavior. With regard to spinal cord injury, these scars were first thought to be a barrier to recovery, but later studies revealed that these scars may be essential in preventing the spread of injury (Yang et al., 2020). Some studies have shown that scars can be modulated through a variety of pharmacological manipulation such as the inhibition of periostin (an extracellular matrix molecule) or inhibition of CSPGs to prevent the formation of scars (Yokota et al., 2017; Yang et al., 2020). Application of these inhibitors during early epileptogenesis combined with antiseizure drugs and/or disease-modifying agents could minimize undesired effects of these scars following DFP induced SE. In summary, this is the first study to characterize glial scars following chemoconvulsant induced SE in the rat DFP model.

## Data Availability

The raw data supporting the conclusion of this article will be made available by the authors, without undue reservation.
