# Supplementary material for: Characterization of Cortical Glial Scars in the Diisopropylfluorophosphate (DFP) Rat Model of Epilepsy
Source: Front Cell Dev Biol. 2022 Mar 16;10:867949. doi: 10.3389/fcell.2022.867949 (PMC8966428; doi:10.3389/fcell.2022.867949)
Supplement: Supplementary file 1 [file Table1.DOCX]

| Antibody | Full name | Dilution | Catalog number | RRID |
| --- | --- | --- | --- | --- |
| Mouse anti-GFAP | Glial fibrillary acidic protein | 1:400 | AB5804 | AB_2109645 |
| Rabbit anti-GFAP | Glial fibrillary acidic protein | 1:400 | NB300141 | AB_10001722 |
| Goat anti-IBA1 | Ionized calcium binding adaptor molecule | 1:300 | Ab5087 | AB_2224402 |
| Rabbit anti-CD68 | Cluster of differentiation 68 | 1:300 | Ab125212 | AB_10975465 |
| Rabbit anti-NeuN | Neuronal nuclei | 1:400 | ABN78 | AB_10807945 |
| Rabbit anti-TFGβ1 | Transforming growth factor 1 | 1:800 | 21898-1-AP | AB_2811115 |
| Rabbit anti-TGβ2 | Transforming growth factor 2 | 1:800 | PA5112839 | AB_2867573 |
| Mouse anti-CS-56 | Chondroitin sulfate | 1:50 | C8035 | AB_476879 |
| Mouse anti-Thy1.1 | Thymocyte differentiation antigen 1 | 1:500 | M7898 | AB_477242 |
| Rat anti-C3 | Complement C3 | 1:150 | NB200-540 | AB_10003444 |
| Rabbit anti-iNOS | Inducible nitric oxide synthase | 1:100 | AB-15323 | AB_301857 |
| Alexaflour 488 anti-mouse |  | 1:80 | NC0192065 | [AB_2340846](http://antibodyregistry.org/AB_2340846) |
| Alexaflour 448 anti-rat |  | 1:80 | A21208 | AB_141709 |
| FITC anti-mouse | Fluorescein isothiocyanate | 1:80 | 711-095-152 | AB_2315776 |
| Biotinylated anti-rabbit |  | 1:400 | **711-065-152** | AB_2340593 |
| Biotinylated anti-mouse |  | 1:400 | **715-065-150** | AB_2307438 |
| Biotinylated anti-goat |  | 1:400 | 705-065-147 | AB_2340397 |
| Cy3 streptavidin |  | 1:300 | **016-160-084** | AB_2337244 |
