# Supplementary material for: Characterization of Cortical Glial Scars in the Diisopropylfluorophosphate (DFP) Rat Model of Epilepsy
Source: Front Cell Dev Biol. 2022 Mar 16;10:867949. doi: 10.3389/fcell.2022.867949 (PMC8966428; doi:10.3389/fcell.2022.867949)
Supplement: Supplementary file 2 [file Table2.DOCX]

**Table S1.** Cell profiler pipeline specifications for immunopositive cell quantification.

| **Marker** | | **Magnification** | | **Diameter min (pixels)** | **Diameter max (pixels)** | **Thresholding strategy** | **Thresholding**  **method** |
| --- | --- | --- | --- | --- | --- | --- | --- |
| IBA1 | 10X | | 9 | | 300 | Global | Otsu |
| CD68 | 10X | | 10 | | 300 | Adaptive | Otsu |
| NeuN | 10X | | 9 | | 300 | Global | Otsu |
| GFAP | 20X | | 26 | | 100 | Global | Otsu |
| C3d | 20X | | 18 | | 100 | Global | Otsu |
| TGFβ1 | 10X | | 9 | | 300 | Global | Otsu |
| TGFβ2 | 10X | | 9 | | 300 | Global | Otsu |
| CS-56 | Counted manually using Image J due to high background | | | | | | |
| iNOS | Counted manually using Image J due to high background | | | | | | |
